# Care Transitions Program for Surgical Patients With Older Age, Cognitive Decline and/or Frailty

**DOI:** 10.1093/geroni/igaf122.4083

**Published:** 2025-12-31

**Authors:** Marianne Desir, Lisa Hue, Sofia Economidou, Naixin Kang

**Affiliations:** Veterans Health Administration, Miami, Florida, United States; Hackensack Meridian Health, North Bergen, New Jersey, United States; University of Miami/Jackson Health System Fellowship in Geriatric Medicine, Miami, Florida, United States; Miami Veterans Affairs Healthcare System, Miami, Florida, United States

## Abstract

C-TraC (Coordinated Transitional Care) is a protocol-driven hospital care transition program that has been offered primarily to patients admitted to medical wards. We adapted C-TraC with a target population of surgical patients with frailty, older age and/or cognitive impairment. A nurse case manager collaborated with a geriatrician and the surgery service to provide pre-discharge visits and weekly post-discharge telehealth visits for up to four weeks. In these visits the nurse: 1) reinforced discharge education; 2) ensured awareness of follow-up appointments; 3) discussed home medications and medication adherence; 4) ensured availability of sufficient functional supports; and 5) ensured awareness of contacts for future questions. After initial piloting in 2023, 51 patients participated in the program during the revised implementation in 2024. Average age was 75 years. Most common surgery types were orthopedic (n = 13), general surgery/surgical oncology (n = 9), vascular (n = 6) and urology (n = 5). 30 patients completed the full C-TraC program. Among historical controls, the readmission rate was 13.3%. Among the 51 patients completing at least the C-TraC visit at hospital discharge, readmission rate was 9.8%. Among the 30 patients who completed the full C-TraC program, the readmission rate was 10.0%. The 30 patients who completed the full program were reached by phone for program evaluation; all described that the program was either moderately or very much a positive aspect of their experience with VA healthcare. Our experience supports that C-TraC can be successfully adapted and implemented for the target population of surgical patients with frailty, older age and/or cognitive impairment.

